# Phenotypic Presentation of Atypical Familial Partial Lipodystrophy in a Patient With a Rare Mutation in the *LMNA* Gene: A Case Report and Literature Review

**DOI:** 10.1155/crie/8775312

**Published:** 2026-11-14

**Authors:** Rebecca Zerbinatti Pereira, Ylana Walleska Santos Santana, Gabriel Monti, Virgínia Oliveira Fernandes, Renan Magalhães Montenegro, Lenita Zajdenverg, Carlos Antonio Negrato

**Affiliations:** ^1^ Graduate Program in Rehabilitation Sciences Concentration Area: Interdisciplinary Care in Different Life Cycles Bauru School of Medicine University of São Paulo (USP) Bauru São Paulo Brazil usp.br; ^2^ Medical Course Bauru School of Medicine University of São Paulo (USP) Bauru São Paulo Brazil usp.br; ^3^ Medical Residency in Family and Community Medicine Albert Einstein Israelite Faculty São Paulo São Paulo Brazil; ^4^ Department of Community Health School of Medicine Federal University of Ceará (UFC) Fortaleza Ceará Brazil ufc.br; ^5^ Clinical Research Unit Walter Cantídio University Hospital Federal University of Ceará/EBSERH Fortaleza Ceará Brazil ufc.br; ^6^ Brazilian Group for the Study of Inherited and Acquired Lipodystrophies (BRAZLIPO) Fortaleza Ceará Brazil; ^7^ Department of Clinical Medicine School of Medicine Federal University of Ceará (UFC) Fortaleza Ceará Brazil ufc.br; ^8^ Department of Internal Medicine Faculty of Medicine Federal University of Rio de Janeiro (UFRJ) Rio de Janeiro Rio de Janeiro Brazil ufrj.br; ^9^ Department of Medicine Bauru School of Medicine University of São Paulo (USP) Bauru São Paulo Brazil usp.br

**Keywords:** case reports, familial partial, inborn errors, laminopathies, lipid metabolism, lipodystrophy, MeSH, progeria

## Abstract

**Background:**

Variants in the *LMNA* gene are classically associated with familial partial lipodystrophy type 2 (FPLD2/Dunnigan syndrome). However, certain mutations produce severe multisystemic phenotypes that may mimic congenital generalized lipodystrophy (CGL). This case adds to the literature by delineating the distinct syndromic expression of the *LMNA* p.Arg349Trp variant and underscoring the need for early recognition to guide cardiovascular and renal surveillance.

**Clinical Findings:**

A 41‐year‐old woman presented to a specialized diabetes clinic serving low‐income populations with severe lipoatrophy of the face and limbs, relative preservation of truncal adiposity, refractory diabetes, severe hypertriglyceridemia, arterial hypertension, and heart failure. Due to the pattern of lipodystrophy, severe systemic involvement, and absence of compensatory cervicofacial fat accumulation, CGL (Berardinelli–Seip syndrome) was initially suspected.

**Diagnoses, Interventions and Outcomes:**

Next‐generation sequencing identified a heterozygous pathogenic *LMNA* variant, c.1045C>T (p.Arg349Trp). At baseline, the patient presented with established proteinuric nephropathy, severe hypertriglyceridemia, and cardiovascular disease. Following multidisciplinary care and an optimized pharmacological protocol, transient improvement in metabolic and glycemic profiles was achieved. However, despite good therapeutic response, socioeconomic barriers disrupted access to high‐cost medications, and she deteriorated to cardiovascular and hepatic failure, dying from cardiogenic shock and sepsis of abdominal origin in less than 2 years of follow‐up.

**Conclusion:**

This case highlights the high clinical severity of the *LMNA* p.Arg349Trp variant. Recognizing it as a distinct syndromic entity is crucial to prevent diagnostic delays, enabling timely cardiovascular and renal surveillance, particularly in resource‐limited settings where treatment interruptions can have fatal consequences.

## 1. Introduction

Lipodystrophies are rare disorders characterized by partial or generalized loss of adipose tissue and can be classified according to etiology (genetic or acquired) or clinical phenotype (generalized or partial). Four major forms are recognized: congenital generalized lipodystrophy (CGL), acquired generalized lipodystrophy, familial partial lipodystrophy (FPLD), and acquired partial lipodystrophy [1–3]. CGL is typically inherited in an autosomal recessive manner and is typically associated with pathogenic variants in *AGPAT2* and *BSCL2* [2–4]. In contrast, FPLD encompasses several inherited variants traditionally classified into distinct subtypes (FPLD1 to FPLD7) based on their genetic etiology [5, 6]. Among these, FPLD2, caused by pathogenic variants in the *LMNA* gene, is the most frequent and well‐characterized form, whose classic phenotype is known as Dunnigan syndrome [6, 7].

The *LMNA* gene, located on chromosome 1q21–22, encodes nuclear lamins A and C, structural proteins essential for nuclear integrity and adipocyte differentiation [8, 9]. Pathogenic *LMNA* variants cause laminopathies, a heterogeneous group of disorders including FPLD‐2, neuropathies, cardiomyopathies, muscular dystrophies, and progeroid syndromes [10]. Among the latter, typical forms such as Hutchinson–Gilford progeria syndrome (HGPS) and mandibuloacral dysplasia (MADA) are well defined, whereas atypical progeroid syndromes (APS) comprise a broader and more variable spectrum of premature aging features, often accompanied by systemic involvement [5].

Recently, the heterozygous *LMNA* p.Arg349Trp variant has been increasingly recognized as driving a distinct, severe phenotype that diverges from classic Dunnigan‐type FPLD2, straddling the spectrum between an atypical FPLD2 and an APS. Case series published by Hussain et al. [11] and Magno et al. [12] describe a characteristic constellation of findings: partial lipodystrophy with predominant loss of fat in the face and extremities, proteinuric nephropathy, frequently focal segmental glomerulosclerosis (FSGS), cardiac disease, metabolic dysfunction, and sensorineural hearing loss. Due to this multisystemic presentation, the term multisystem progeroid syndrome (MSPS) has been proposed for individuals harboring this specific variant [11].

We report a patient followed in a diabetes clinic whose severe phenotype initially mimicked CGL. Molecular analysis, however, identified the *LMNA* p.Arg349Trp variant, and a targeted literature review demonstrated marked compatibility with the MSPS phenotype. This case contributes to the growing body of evidence delineating the clinical spectrum and severity associated with this rare genotype. Moreover, it documents the rapid clinical progression and fatal outcome of this condition within a resource‐limited, volunteer‐based healthcare setting, further complicated by an absence of a family history due to adoption. This case underscores the vital necessity of early phenotypic recognition to bypass diagnostic bottlenecks and guide timely cardiovascular and renal interventions.

## 2. Case Presentation

We report the case of a patient followed at our multidisciplinary diabetes clinic for low‐income patients from June 2022 to March 2024. She first presented to our service at 41 years of age with complaints of polyuria, pollakiuria, polydipsia, involuntary weight loss, asthenia, and chronic constipation. She had no hearing‐related symptoms.

Six years earlier, she had been diagnosed with diabetes mellitus (DM), systemic arterial hypertension (SAH), and dyslipidemia. She also reported a history of coronary artery disease (CAD) and heart failure with preserved ejection fraction (HFpEF), for which she was receiving follow‐up with a cardiologist at another outpatient clinic, although no confirmatory complementary exams were presented at the time of admission to our service. Her family history was entirely unknown as she was adopted, which limited the assessment of inherited conditions or familial cardiac events.

At baseline, her daily pharmacological regimen included NPH insulin at 0.96 IU/kg (20 IU morning, 3 IU afternoon, and 10 IU night), regular insulin at 0.80 IU/kg (14 IU before lunch and 18 IU before dinner), alogliptin 25 mg + pioglitazone 30 mg, metformin 2 g, gliclazide MR 120 mg, simvastatin 40 mg, acetylsalicylic acid 100 mg, metoprolol 50 mg, spironolactone 50 mg, furosemide 80 mg, and digoxin 0.25 mg.

### 2.1. Clinical Assessment

On physical examination, she presented with a complex phenotype of partial lipodystrophy, characterized by a marked reduction in subcutaneous adipose tissue in the face, buttocks, and limbs, more pronounced in the lower than in the upper extremities. There was mild preservation of subcutaneous adipose tissue in the thoracic and abdominal regions. Also, residual adipose tissue was present in the pubic, infraaxillary, and mammary regions, although the breast volume was reduced. The extremities were thin and elongated, with prominent skeletal musculature and no evidence of muscle hypotrophy.

The patient also had a narrow face, prominent zygomatic arches, a tapered jaw, thin lips, a hooked nose, mild micrognathia, and diffuse loss of facial subcutaneous fat, with preservation of adipose tissue in the orbital region, scalp, and oral cavity. The skin was thin and adherent, with prominent superficial veins in both the upper and lower limbs, without lipid deposits or cutaneous lesions such as *Acanthosis nigricans*.

Anthropometric measurements were obtained. The patient had a slightly short stature (1.56 m). Throughout follow‐up, body weight ranged from 39.4 to 41.1 kg, and BMI varied only slightly between 16.19 and 16.89 kg/m^2^, consistently indicating underweight status. The waist‐to‐hip ratio remained between 0.93 and 0.95 during most of the follow‐up, increasing to 1.01 only at the final evaluation in March 2024, concomitant with marked clinical deterioration.

Cardiovascular examination revealed a systolic ejection murmur. Abdominal examination showed hepatomegaly, with the liver palpable 3 cm below the right costal margin and a liver span of 14 cm.

Figure 1 illustrates the findings as described. Based on this clinical pattern, the initial diagnostic suspicion recorded in the medical record was Berardinelli–Seip syndrome (a form of CGL). Written informed consent was obtained from the patient for the use of her clinical, biochemical, molecular, and photographic data in the case report. The case report was approved by the Institutional Research Ethics Committee (REC).

**Figure 1:**
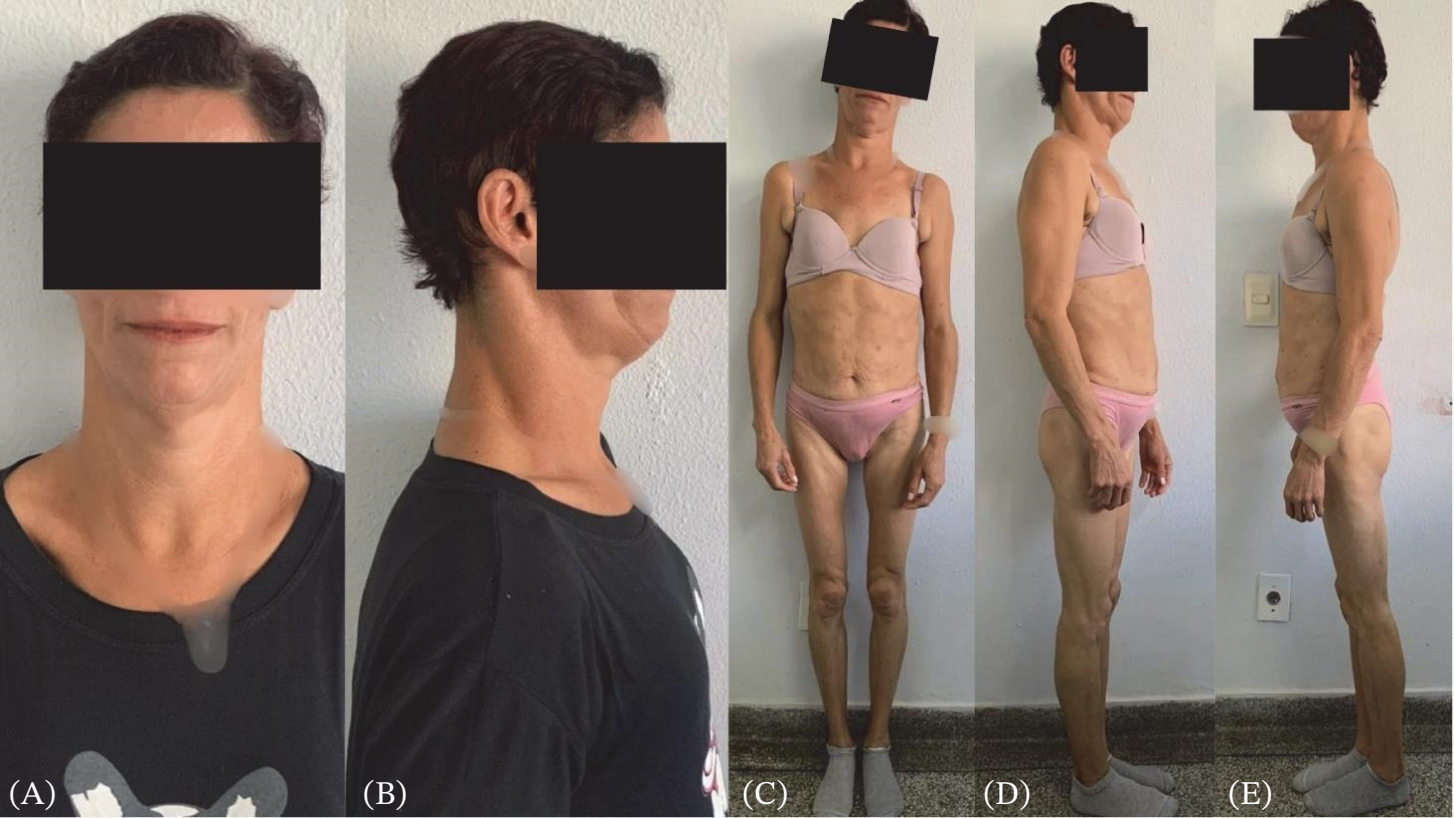
Clinical aspect of the patient with partial lipodystrophy: (A) head and neck, frontal view; (B) head and neck, right lateral view; (C) whole body, frontal view; (D) whole body, right lateral view; (E) whole body, left lateral view.

### 2.2. Molecular Analysis of the *LMNA* Gene

Subsequently, molecular analysis was initiated to confirm the clinical suspicion of lipodystrophy. DNA was extracted from a buccal swab and sent to the laboratory for next‐generation sequencing using the Illumina technology. The files were processed using the ExomeDepth program, and gnomAD was used for the allelic frequency reference of the variants. Genetic analysis identified the heterozygous variant c.1045C>T (p.Arg349Trp) in the *LMNA* gene.

### 2.3. Laboratory Evaluation and Complementary Tests

Routine biochemical analyses were requested at baseline and every 3 months to monitor the metabolic status. Venous blood samples were collected after a 12‐h fast to include the glycemic profile, lipid profile, and renal function, in addition to measuring the albumin‐to‐creatinine ratio (ACR) in a single urine sample. The laboratory data are shown in Table 1.

**Table 1:** Evolution of laboratory tests during follow‐up. Prepared by the authors.

Laboratory test	2022/06	2022/09	2022/12	2023/03	2023/06	2023/09	2023/11
HbA1c (%)^a^	10.2	7.9	7.0	7.2	7.7	7.2	7.5
Fasting glycemia (mg/dL)	141.3	124.2	89.2	156.0	181.3	93.5	100.5
Total cholesterol (mg/dL)	192	191	166	176	168	141	169
HDL‐cholesterol (mg/dL)^b^	29	36	35	31	35	30	31
LDL‐cholesterol (mg/dL)^c^	110	119	96	108	96	78	112
Triglycerides (mg/dL)	373	254	239	241	258	234	150
Creatinine (mg/dL)	0.91	0.93	0.95	0.79	0.80	1.13	0.94
Albumin/creatinine ratio (mg/g)	1422	3248	2241	2900	4003	4207	2.0

*Note: Source:* Patient’s physical medical record.

^a^HbA1c: glycated hemoglobin.

^b^HDL: high‐density lipoprotein.

^c^LDL: low‐density lipoprotein.

Complementing routine laboratory tests, a fundus examination was performed in July 2022, which revealed mild diabetic retinopathy in both eyes. Furthermore, advanced diagnostic imaging modalities, such as dual‐energy X‐ray absorptiometry (DEXA) and magnetic resonance imaging (MRI), were unavailable in this clinical setting.

### 2.4. Management Actions

Therapeutic management combined lifestyle interventions with pharmacological protocols. The interdisciplinary team continuously reinforced diabetes self‐management education following the guidelines of the Brazilian Diabetes Society (SBD), focusing on dietary adherence, strict dosing schedules, correct insulin injection technique, site rotation, and proper storage handling. Intensive capillary glucose monitoring at key time points throughout the day (fasting before lunch and before dinner), supplemented by bedtime measurements, was implemented to optimize insulin therapy. This approach was complemented by biweekly multidisciplinary consultations to assess clinical stability and treatment adherence.

Dietary counseling, guided by a nutritionist, emphasized a low‐fat, low‐sodium, and low‐glycemic index regimen to manage severe hypertriglyceridemia and insulin resistance. Over the follow‐up period, dietary adherence was variable. Although the patient made significant efforts to adhere to dietary recommendations, strict compliance was hindered by her routine work in the kitchen and episodes of anxiety‐related compulsive eating.

Given the patient’s underlying cardiovascular disease, regular physical activity was cautiously recommended and monitored by a cardiologist at an external cardiology outpatient clinic, limited to low‐to‐moderate‐intensity exercises to optimize metabolic control while safeguarding cardiac reserve. However, as the underlying cardiovascular disease progressed, she developed exertional dyspnea, nocturnal orthopnea, and severe fatigue. These symptoms forced a progressive reduction of her physical activity, eventually leading to marked exercise intolerance and a complete cessation of physical exercise.

Pharmacological management focused on escalating insulin doses and optimizing lipid‐lowering therapy. Basal insulin doses were finely adjusted to 0.93 IU/kg/day of NPH insulin (22 IU morning, 10 IU afternoon, and 6 IU at bedtime), combined with mealtime sliding‐scale rapid‐acting insulin (up to 18 IU/dose). This basal insulin regimen was redistributed due to recurrent nocturnal hypoglycemia episodes. Additionally, lipid‐lowering treatment was optimized by switching simvastatin to atorvastatin 40 mg/day and adding fenofibrate 200 mg/day, while enalapril 20 mg/day was introduced to provide renal protection and optimize blood pressure control.

Although this targeted regimen achieved initial metabolic improvement, treatment continuity was severely compromised by public health supply shortages and socioeconomic vulnerability. Stockouts at the high‐cost public pharmacy network repeatedly forced the discontinuation of atorvastatin, fenofibrate, and pioglitazone/alogliptin, as well as the substitution of rapid‐acting insulin analogs with regular insulin.

### 2.5. Evolution

After 6 months of follow‐up, partial improvement in the lipid and glycemic profile was observed (Table 1), whereas anthropometric parameters remained essentially unchanged.

Despite regular follow‐up and good adherence to treatment, cardiac and hepatic impairment progressively worsened. At her final outpatient visit in March 2024, the patient presented with New York Heart Association (NYHA) Class IV heart failure, characterized by dyspnea at rest, nocturnal cough, and markedly reduced exercise tolerance. Although she remained on optimized therapy with ACE inhibitors, beta‐blockers, diuretics, and digoxin, management was hindered by socioeconomic barriers. Interruption of outpatient follow‐up delayed the renewal of medications supplied through the public high‐cost pharmacy program, leading to the discontinuation of atorvastatin and fenofibrate. An episode of acute febrile gastroenteritis ~15 days before the final visit likely served as an additional infectious stressor contributing to clinical decompensation.

In late March 2024, the patient was hospitalized for an abdominal infection associated with cardiac and hepatic decompensation, resulting in death. The recorded causes of death were cardiogenic shock, ventricular tachycardia, sepsis of abdominal origin, bacterial peritonitis, hepatic cirrhosis, and heart failure.

## 3. Discussion

The present case describes a patient carrying the p.Arg349Trp (c.1045C>T) variant in the *LMNA* gene [13]. As *LMNA* gene variants underlie FPLD2, Dunnigan syndrome represents its classical and best‐characterized phenotype. However, while it is typically characterized by progressive loss of subcutaneous adipose tissue from the limbs and trunk with relative fat accumulation in the cervicofacial region [1, 5, 6], the phenotype observed in this patient differed from this pattern. Instead, the patient presented with profound limb lipoatrophy and additional multisystem manifestations that also raised the initial suspicion of CGL, despite not fulfilling its typical phenotype. The absence of family history due to adoption further complicated the diagnostic process by eliminating important clues, such as familial cardiomyopathy, sudden cardiac death, or consanguinity.

### 3.1. Molecular Basis

From a molecular perspective, the distinctive phenotype associated with p.Arg349Trp may be related to its specific location within the *LMNA* protein. While most FPLD2‐associated variants occur in the C‐terminal globular domain and affect adipogenic pathways through altered protein interactions, p.Arg349Trp is located in the central alpha‐helical rod domain of lamin A/C, a region essential for dimer formation and interaction with nuclear proteins [9]. This substitution of a highly conserved arginine by tryptophan is predicted to modify the electrostatic properties of the rod domain and impair interactions required for normal nuclear function, providing a possible explanation for the multisystemic phenotype observed in these patients [14].

Although the mechanisms underlying the severe phenotype associated with p.Arg349Trp remain incompletely understood, studies of other *LMNA*‐related lipodystrophies have revealed pathogenic pathways that may contribute to adipose dysfunction and metabolic complications. Adipose tissue‐specific models demonstrated that mutant lamins promote extracellular matrix remodeling and fibrosis, reducing adipocyte expandability and favoring ectopic lipid deposition through mechanisms involving TGF‐β signaling [15]. This impaired capacity for lipid storage contributes to metabolic inflexibility and the accumulation of toxic lipid intermediates in nonadipose tissues, potentially leading to complications such as hepatic steatosis, insulin resistance, and myocardial dysfunction [9]. Consistent with these mechanisms, clinical studies have shown that reduced lower‐limb fat mass is associated with higher triglyceride levels and increased risk of severe organ complications, including nonalcoholic steatohepatitis and cardiac dysfunction [16].

### 3.2. Topography of Fat Loss and Phenotypic Markers

As detailed in recent case series by Hussain et al. [11] and Magno et al. [12], individuals harboring the p.Arg349Trp variant show a distinct pattern of lipodystrophy: fat loss in the face, extremities, and, characteristically, in the palms of the hands and soles of the feet. Fat loss in the limbs is often described as more pronounced distally (forearms and calves) than proximally (arms and thighs). This specific pattern, including micrognathia, was previously documented by Mory et al. [8] in a young patient carrying the exact same mutation, reinforcing the clinical consistency of this genotype.

Although physical examination indicated relative truncal fat preservation in our patient, truncal fat distribution in p.Arg349Trp carriers is highly variable. While the literature often describes preserved or increased abdominal fat in these patients, this is not a universal rule. Notably, patient MSPS 300.3 from the study by Hussain et al. [11] presented with apparent abdominal fat on physical examination, yet DEXA analysis exposed severe total (12.7%) and truncal (14.4%) fat depletion.

For an objective evaluation of this regional fat loss, Ajluni et al. [16] suggest the use of DEXA to calculate the fat mass ratio (FMR), which is the ratio of trunk fat percentage to leg fat percentage. By applying this ratio to their cohort and comparing it with control groups, they demonstrated that an FMR cutoff above 1.5 serves as a highly predictive diagnostic marker for FPLD in adult females, achieving a sensitivity of 86.7% and a specificity of 96.8%. Furthermore, recent literature shows that high‐precision methods such as DEXA and MRI can reveal subclinical changes in fat deposition that escape physical examination [6].

### 3.3. Multisystemic Involvement

The precise identification of this syndrome is fundamental because its clinical impact extends far beyond the topographical distribution of the adipose tissue. Renal involvement typically manifests as severe proteinuric nephropathy, often FSGS [11, 12]. Thong et al. [14] demonstrated clear familial cosegregation of biopsy‐proven FSGS with the p.Arg349Trp variant. As highlighted by Fountas et al. [17], while proteinuric renal disease is a prevalent feature in generalized lipodystrophies, it is exceptionally rare in familial partial lipodystrophies, rendering its presence a major diagnostic hallmark of this specific mutation.

Our patient presented progressive and severe albuminuria, reaching nephrotic‐range levels (Table 1). In interpreting these findings, a multifactorial etiology must be considered. Although diabetes and hypertension are established contributors to renal disease, her severe albuminuria was markedly disproportionate to her mild diabetic retinopathy. This suggests that the *LMNA* mutation potentially acted as a primary driver or potent accelerator of podocyte injury.

Cardiovascular involvement represents a critical determinant of the prognosis. As highlighted by Magno et al. [12], cardiovascular complications are highly penetrant in p.Arg349Trp carriers, manifesting as arrhythmias (81%), valvular abnormalities (62%), and cardiomyopathy. Our patient’s history of CAD, HFpEF, systolic murmur, and fatal progression to ventricular tachycardia and cardiogenic shock closely aligns with these reports. The aggressiveness of these terminal events is consistent with structural findings reported by Bhaskaran et al. [18], whose multicenter cohort analysis demonstrated that missense variants located in the *LMNA* alpha‐helical rod domain have significantly worse cardiac prognoses compared to variants in the tail domain. Specifically, rod domain variants carry a nearly threefold higher annual event rate for malignant ventricular arrhythmias and confer a risk of advanced heart failure that closely approaches that of truncating mutations[18].

Additionally, severe insulin resistance, difficult‐to‐control DM, and marked hypertriglyceridemia are consistently observed [11, 12, 19]. Sensorineural hearing loss, reported in ~66% of p.Arg349Trp carriers [11, 12], was not formally tested in our patient as no subjective auditory symptoms were reported.

### 3.4. Therapeutic Considerations and Disease Course

The therapeutic management currently relies on a multidisciplinary approach focused on mitigating its severe metabolic, renal, and cardiovascular complications [1, 6, 10]. For the p.Arg349Trp variant specifically, evidence is currently limited to single‐case reports, in which a combination of metformin, fenofibrate, and the GLP‐1 analog liraglutide was shown to improve metabolic profiles and reduce visceral fat [19]. Furthermore, rigorous renal and cardiovascular monitoring is crucial [1, 5], often necessitating the use of ACE inhibitors to manage proteinuria [17, 19] and the early placement of implantable cardioverter‐defibrillators or pacemakers to prevent life‐threatening arrhythmias and sudden cardiac death [10, 20, 21]. Also, recombinant human methionyl leptin (metreleptin) represents a therapeutic option for selected patients with *LMNA*‐associated FPLD who exhibit severe, refractory metabolic derangements or marked hypoleptinemia [1, 5, 10, 16].

Multisystemic manifestations associated with this specific genotype are exceptionally severe, contributing to high morbidity and reduced life expectancy. In carriers of the p.Arg349Trp variant, premature death in the third or fourth decade of life is frequent, with the literature reporting fatal outcomes from heart failure, malignant arrhythmias, and multiorgan failure [11, 12, 22]. This devastating clinical course can be best understood when analyzed alongside the mortality patterns of other *LMNA*‐associated disorders. In classic FPLD, life expectancy is reduced due to chronic metabolic burden, but patients generally reach an older age (mean survival ~66.6 years; 33% cardiovascular mortality) [7]. Conversely, in progeroid syndromes such as HGPS, accelerated atherosclerosis leads to premature death in childhood (mean age 13.4–14.6 years) [9, 23, 24]. The MSPS phenotype bridges these distinct entities by combining the severe metabolic dysregulation of FPLD, the accelerated vascular aging characteristic of progeroid syndromes, and the aggressive arrhythmogenic cardiomyopathy typical of rod‐domain laminopathies [9, 11, 12, 18, 25].

Ultimately, this specific genotypic risk corroborates the catastrophic cardiovascular outcome observed in our patient, closely aligning with previous reports of sudden cardiac death in the fourth decade of life among other individuals harboring this exact variant [11, 22, 26]. However, it is hypothesized that external factors may have influenced its course. Socioeconomic vulnerability and restricted access to high‐complexity healthcare may be factors that contribute to the faster progression of complications. While it is difficult to determine whether these factors were the primary drivers, they represent critical variables that should be considered in the present case. Nonetheless, recent literature supports the concept that external triggers can precipitate rapid deterioration in this vulnerable population; for instance, Ceccarini et al. [27] described the onset of severe postacute cardiac complications following a viral infection (SARS‐CoV‐2) in a patient harboring the same p.Arg349Trp variant. In our patient, the recent gastroenteritis episode, compounded by the forced discontinuation of lipid‐lowering therapy due to health system barriers, likely accelerated the multiorgan failure.

### 3.5. Study Limitations

A significant limitation of this report is the absence of specialized diagnostic data, such as serum leptin and adiponectin levels, which were unavailable at our regional center. Furthermore, the patient’s rapid clinical decline, compounded by restricted access to medical records regarding her final hospitalization, limited a more thorough exploration of the immediate causes of mortality. These factors also precluded the performance of a renal biopsy as well as high‐precision body composition imaging, such as DEXA or MRI. The analysis of the body fat distribution was based on visual inspection. This approach, although practical, is subject to potential observer bias and limits the accuracy of body composition data.

While we acknowledge the potential for observer bias and the subjective nature of this assessment, it reflects a common diagnostic challenge in resource‐limited settings. In such contexts, recognizing the MSPS phenotype remains a crucial step for early genetic counseling and the management of potentially fatal multisystem complications, even in the absence of specialized biomarkers.

In conclusion, our patient occupied a diagnostic intersection: she was classified as both atypical lipodystrophy and an APS. Given the consistency of this high‐severity profile, the broad label of “atypical” is no longer clinically sufficient. Instead, we advocate for the classification of the phenotype caused by the *LMNA* p.Arg349Trp variant, as proposed by Hussain et al. [11]. Recognizing MSPS as a unique entity shifts the focus from a variable fat distribution to an aggressive syndrome. While classic FPLD cohorts exhibit a mean overall survival of ~66.6 years, this specific genotype drives a devastating natural history, precipitating fatal cardiogenic shock, malignant arrhythmias, and multiorgan failure often in the third or fourth decade of life [7, 22]. This extreme severity must be contextualized within the reality of regional or resource‐limited medical centers, where access to high‐precision diagnostic tools is restricted. Under these real‐world conditions, defining this mutation as a distinct syndromic presentation based on its unique physical hallmarks empowers clinicians to bypass diagnostic bottlenecks. Recognizing this specific clinical phenotype should serve as an immediate alarm signal, prompting aggressive cardiovascular and renal screening and interventions, even when advanced metabolic and molecular panels are temporarily unavailable.

## 4. Conclusion

This case report describes a patient with the p.Arg349Trp variant in the *LMNA* gene. Although the severe clinical presentation could mimic GCL, the detailed phenotype (partial lipodystrophy with trunk preservation) and multisystemic complications (proteinuric nephropathy and severe cardiomyopathy) are, in fact, consistent with MSPS recently described in the literature for this specific genotype. The fatal outcome corroborates the documented severity of this variant, reinforcing the importance of molecular diagnosis and review of the variant‐specific literature to differentiate this condition from other laminopathies.

## Author Contributions

Ylana Walleska Santos Santana, Rebecca Zerbinatti Pereira, Gabriel Monti, Lenita Zajdenverg, and Carlos Antonio Negrato participated in the collection of clinical data and the preparation of the manuscript. Ylana Walleska Santos Santana, Rebecca Zerbinatti Pereira, Gabriel Monti, Virgínia Oliveira Fernandes, Renan Magalhães Montenegro, Lenita Zajdenverg, and Carlos Antonio Negrato participated in the data analysis, critical review of the content, and final approval of the version to be published.

## Funding

This work did not receive any public or private funding for its realization.

## Disclosure

All authors have read and approved the final version of the manuscript. The corresponding author, Rebecca Zerbinatti Pereira, had full access to all of the data in this study and take complete responsibility for the integrity of the data and the accuracy of the data analysis. All generated text was thoroughly reviewed, edited, and validated by the authors, who take full responsibility for the accuracy, integrity, and originality of the final manuscript.

## Ethics Statement

This study was conducted in accordance with the ethical principles of the Declaration of Helsinki and was approved by the Institutional Research Ethics Committee (CAAE: 01680226.2.0000.5441). Free and informed consent was obtained from the patient for the use of clinical, laboratory, imaging, and genetic data, as well as for the publication of information and images related to the case, ensuring anonymity and confidentiality.

## Conflicts of Interest

The authors declare no conflicts of interest.

## Data Availability

Data that support the findings of this study are available in the article itself. Due to the nature of this research (case report) and to protect patient privacy, additional raw data are not publicly available but may be available from the corresponding author upon reasonable request.
